# Lived experiences of ethical dilemmas in pricing life-saving medicines among private-sector pharmacists in Zimbabwe

**DOI:** 10.4102/phcfm.v18i1.5205

**Published:** 2026-02-04

**Authors:** Daniel Sibanda

**Affiliations:** 1 Graduate Business School, University of Zambia, Lusaka, Zimbabwe; 2 Department of Biochemistry and Pharmacology, Faculty of Medicine, National University of Science and Technology, Bulawayo, Zimbabwe

**Keywords:** pharmacy ethics, medicine pricing, moral distress, access to medicines, Zimbabwe, primary health care

## Abstract

**Background:**

Access to affordable medicines is central to primary health care and universal health coverage. In Zimbabwe, the absence of national price regulation and heavy out-of-pocket expenditure place private-sector pharmacists at the centre of difficult decisions, as they balance professional duty with business survival.

**Aim:**

This study aimed to explore the ethical dilemmas faced by pharmacists in Zimbabwe’s private sector when pricing life-saving medicines and the coping mechanisms and support systems they employ.

**Setting:**

The study was conducted in private community pharmacies located in Harare and Bulawayo, Zimbabwe’s two largest urban pharmaceutical markets.

**Methods:**

A qualitative interpretive phenomenological design was used. Semi-structured interviews were conducted with 12 pharmacists (6 Harare, 6 Bulawayo) who were purposively sampled from independent and chain pharmacies. Interviews were recorded, transcribed verbatim, and thematically analysed using Braun and Clarke’s six-phase framework. NVivo supported coding.

**Results:**

Pharmacists described moral distress when patients could not afford medicines, particularly insulin and antihypertensives. Informal coping strategies included silent discounts, partial dispensing, referrals, and credit sales. Harare participants emphasised currency volatility and chain-store policies, whereas Bulawayo pharmacists highlighted community solidarity. All called for systemic support through ethical pricing guidelines, subsidy mechanisms, and professional forums.

**Conclusion:**

Ethical dilemmas in pricing are pervasive. Coping strategies provide temporary relief but are unsustainable and may compromise care. Systemic reforms are required to balance affordability with sustainable pharmacy practice.

**Contribution:**

This study highlights the moral dimension of medicine pricing in Zimbabwe and provides evidence to guide ethical frameworks, regulatory reforms, and professional support for equitable access to medicines.

## Introduction

Access to affordable, quality medicines is a cornerstone of primary health care (PHC) and universal health coverage (UHC).^1^ Yet, in many low- and middle-income countries (LMICs), including Zimbabwe, the private retail pharmacy sector plays a critical role in medicine supply amid fragile public systems.^2^ In this environment, pharmacists function simultaneously as clinicians and business operators, navigating the competing imperatives of patient welfare and commercial sustainability. The absence of national price regulation for medicines in Zimbabwe exacerbates this tension, often leaving pharmacists to determine prices in a volatile market shaped by foreign currency shortages, inflation, and import dependence.^3^

From a public health perspective, medicine pricing practices directly influence access to essential therapies for chronic and life-threatening conditions such as hypertension, diabetes, and human immunodeficiency virus (HIV) and/or acquired immunodeficiency syndrome (AIDS). Studies across Africa and Asia have shown that unregulated pricing frequently results in unaffordable mark-ups, stock-outs, and inequities in access between rich and poor communities.^4,5,6^ In Zimbabwe, over 70% of health expenditures are out-of-pocket, creating financial hardship for households and moral dilemmas for pharmacists when patients cannot afford prescribed treatments.^7,8,9^

From a scientific perspective, there is a growing body of literature examining pharmaceutical ethics and affordability in LMICs, but relatively little has explored the lived experiences of pharmacists in Zimbabwe’s private sector.^3,4^ Research elsewhere has highlighted moral distress when health professionals must deny care because of patients’ inability to pay, yet little is known about how Zimbabwean pharmacists cope with these dilemmas, what informal strategies they employ, and how they perceive their dual obligations to patients and business viability.^8^ This represents a critical knowledge gap in both pharmacy ethics and health systems research in Southern Africa.^2,3^

To address this gap, this study explores the ethical dilemmas faced by private-sector pharmacists in pricing life-saving medicines in Zimbabwe. Specifically, it investigates the internal conflicts, coping mechanisms, and perceived need for systemic or professional support. By giving voice to pharmacy professionals, the study seeks to contribute to ongoing debates on equitable access to medicines and to inform the development of ethical pricing frameworks and supportive policies.^6,10^

### Aim and objectives

The aim of this study was to explore the ethical dilemmas faced by pharmacists in Zimbabwe’s private retail sector in pricing life-saving medicines. The objectives were:

- to describe the nature of ethical conflicts experienced by pharmacists in relation to medicine pricing
- to examine the coping mechanisms pharmacists adopt when confronted with patients unable to afford medicines
- to identify policy and professional support needs to enable ethical and sustainable pricing practices.

## Research methods and design

### Study design

This study employed a qualitative design, drawing on in-depth, semi-structured interviews with private-sector pharmacists. The approach was informed by interpretive phenomenology, aiming to capture the lived experiences of pharmacists as they navigated ethical dilemmas in medicine pricing. This qualitative emphasis complemented broader mixed-methods work conducted in Zimbabwe, which had previously applied the World Health Organization/Health Action International (WHO/HAI) methodology to assess the affordability and availability of essential medicines.

### Study’s setting

The research was conducted in two major urban centres, Harare and Bulawayo, which collectively represent Zimbabwe’s largest pharmaceutical retail markets. Harare, the capital, is the primary hub for pharmaceutical imports, wholesaling, and high-income clientele, while Bulawayo reflects both middle-income and working-class populations, with marked disparities between affluent suburbs (e.g. Burnside, Hillside) and low-income areas (e.g. Makokoba, Mzilikazi). This dual-city focus allowed for exploration of urban inequalities in access to medicines and pricing ethics.

### Study population and sampling

The participants were licensed pharmacists operating in private retail pharmacies. Both independent pharmacies and branches of larger chains were included to capture variability in business models and pricing practices. Pharmacists were eligible if they had at least 2 years’ post-registration experience and were directly involved in pricing or dispensing decisions. Using purposive and snowball sampling, 12 pharmacists (6 from Harare and 6 from Bulawayo) were recruited. This sample size was consistent with qualitative research standards for thematic saturation.^11^

### Data collection

Data were collected between September 2024 and November 2024 through semi-structured face-to-face interviews lasting 45 min to 60 min each. The interview guide was expert-reviewed for content validity and piloted with two pharmacists. The interview guide was designed to probe perceptions of medicine pricing, ethical dilemmas, coping mechanisms, and systemic barriers. Key areas explored included:

- Experiences of pricing life-saving medicines under unregulated conditions.
- Ethical conflicts when patients could not afford treatment.
- Strategies used to balance professional duty and business sustainability.
- Perceptions of regulatory frameworks and policy support.

All interviews were conducted in English and audio-recorded with consent. Field notes were maintained to capture contextual observations.

### Data management and analysis

Interviews were transcribed verbatim and checked for accuracy against audio recordings. Thematic analysis was undertaken following Braun and Clarke’s six-phase framework. Coding was both: inductive, emerging from pharmacists’ narratives and deductive, guided by concepts from regulation theory and health economics. NVivo software was used to support data management, coding, and theme development.

Emergent themes were triangulated with quantitative findings from the broader doctoral research, which included WHO/HAI pricing surveys conducted in Harare and Bulawayo. This triangulation strengthened credibility and provided context for the ethical dilemmas described.

### Ethical considerations

Ethical clearance for this study was obtained from the University of Zambia Biomedical Research Ethics Committee (UNZABREC Ref. No. 5732-2024) and the Medical Research Council of Zimbabwe (MRCZ/A/2647). Permission to access private pharmacies was granted by the Ministry of Health and Child Care of Zimbabwe. Written informed consent was obtained from all participants. Confidentiality and anonymity were strictly maintained.

## Results

### Participants’ characteristics

Participant quotations are identified using participant number, city, and years of professional experience. A total of 12 pharmacists were interviewed (6 in Harare, 6 in Bulawayo). Participants represented both independent pharmacies and branches of large retail chains. The majority had over 5 years of practice experience, with a nearly equal distribution of male and female respondents. Participant characteristics are summarised in Table 1.

**TABLE 1:** Demographic characteristics of interviewed pharmacists.

Variable	Harare (*n* = 6)	Bulawayo (*n* = 6)	Total (*N* = 12)
Male	3	4	7
Female	3	2	5
Pharmacists with > 5 years’ experience	5	4	9
Chain pharmacies	4	3	7
Independent pharmacies	2	3	5

### Theme 1: Ethical conflicts in pricing

Pharmacists consistently described moral distress when patients were unable to afford life-saving medicines. This was especially evident for chronic disease therapies such as insulin and antihypertensives. The recurring theme across interviews was the emotional and ethical strain associated with balancing professional duty to patients and business sustainability in an unregulated pricing environment:

‘I feel torn when a diabetic patient walks away without insulin because the price is beyond their reach. It stays with me long after work.’ (P02, Bulawayo, 5 years)

‘At times I know the patient needs the medicine immediately, but the owner insists we cannot reduce the price. It makes me feel like I am failing as a professional.’ (P11, Harare, 3 years)

‘It’s not just about profit. We live in these communities. When someone’s child needs antibiotics, you can’t just turn them away because they are short by a few dollars.’ (P03, Bulawayo, 7 years)

‘Some patients accuse us of being greedy, but they don’t realise we also buy stock in USD. You feel guilty, but you also have rent and salaries to pay.’ (P12, Harare, 6 years)

‘There are days when I go home questioning if I’m still helping people or just surviving in business.’ (P05, Bulawayo, 12 years)

‘We face emotional burnout. The system pushes us to make choices between empathy and economics every day.’ (P16, Harare, 4 years)

Across both cities, pharmacists expressed that such ethical tension was not limited to expensive medicines but extended to basic drugs such as antibiotics and antihypertensives. Harare pharmacists often reported stronger managerial pressure and less autonomy in pricing decisions, while Bulawayo pharmacists emphasised solidarity and personal relationships with patients – sometimes offering discounts at their own financial expense:

‘If I don’t give a small discount, I may lose a loyal client who has been coming for years. It’s not sustainable, but it feels like the right thing to do.’ (Pharmacist, Bulawayo)

‘Our policies are fixed by the head office; even when I want to help, I cannot change prices on my own.’ (Pharmacist, Harare)

These accounts reflect the multifaceted ethical conflicts pharmacists face – between compassion and compliance, autonomy and accountability, and professional ethics versus financial survival. The moral distress expressed across interviews underscores the urgent need for structural reforms to support ethically grounded pricing decisions.

### Theme 2: Coping mechanisms

Pharmacists employed a range of informal strategies to mitigate patient hardship:

- Silent discounts for repeat or visibly distressed clients.
- Dispensing partial dosages to reduce immediate costs.
- Referrals to cheaper outlets or non-governmental organisation (NGOs).
- Offering credit sales to trusted clients.

These coping practices appeared more commonly described by pharmacists in Bulawayo, who emphasised stronger community solidarity, whereas pharmacists in Harare highlighted greater competitive pressures that constrained their flexibility. The range of coping mechanisms reported by

pharmacists is presented in Table 2.

**TABLE 2:** Common coping mechanisms reported by pharmacists.

Coping mechanism	Harare (*n* = 6)	Bulawayo (*n* = 6)	Illustrative quote
Silent discounts	3	5	‘Sometimes I just knock off a few dollars quietly.’ (P13, Harare, 7 years)
Partial dosages	2	4	‘We give half the course and tell them to return when they get more money.’ (P02, Bulawayo, 5 years)
Referrals to NGOs and/or outlets	4	5	‘I tell them about Mission Hospital dispensary where it is cheaper.’ (P14, Harare, 9 years)
Credit sales	1	3	‘Some trusted patients, we write it down in a book until payday.’ (P04, Bulawayo, 9 Years)

NGOs, non-governmental organisations.

### Theme 3: Variations between Harare and Bulawayo

Significant city-level differences emerged:

- **Harare:** Pricing was driven more by foreign currency fluctuations, competitive business pressures, and chain-store policies.
- **Bulawayo:** Pharmacists described closer relationships with patients, greater willingness to offer informal credit, but also higher stock-out risks.

**FIGURE 1:**
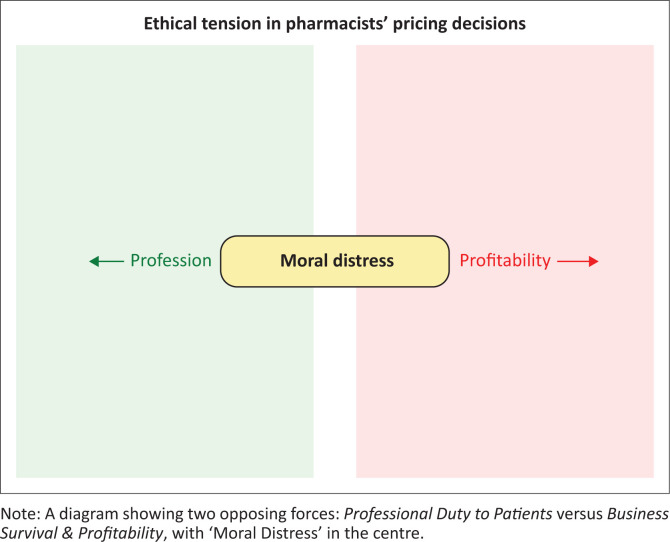
Schematic of ethical tension in pharmacists’ pricing decisions.

Although pharmacists across both cities described similar ethical pressures, their lived experiences highlighted meaningful contextual differences in how these dilemmas were encountered and navigated. These variations were shaped by contrasting economic environments, organisational structures, and community relationships. Figure 1 illustrates the ethical tension between professional duty and business survival experienced by pharmacists.

*Harare pharmacists* commonly spoke about the constraints created by currency volatility, competitive business environments, and chain-store policies that limited their autonomy in pricing decisions:

‘Here in Harare prices change with the rate. Even if I want to help a patient, the system updates the price automatically.’ (P12, Harare, 5 years)

‘Competition is fierce. If you reduce prices too much, you risk being seen as undermining the chain’s business model.’ (P13, Harare, 5 years)

These accounts reflect an environment where commercial pressures and operational policies more strongly restrict the ability to offer informal support to patients.

In contrast, *Bulawayo pharmacists* emphasised closer community relationships and a stronger expectation of social solidarity, which shaped their ethical decision-making:

‘Our patients are like family here. You can’t watch someone walk away without medicine if you know their situation.’ (P01, Bulawayo, 3 years)

‘Sometimes we allow people to take the medicines and pay later because the community depends on us.’ (P03, Bulawayo, 7 years)

However, this sense of solidarity was tempered by frequent stock-outs and cash-flow limitations:

‘You want to help, but stock delays in Bulawayo make it difficult. Sometimes even if you want to give credit, the shelves are empty.’ (P04, Bulawayo, 9 years)

**FIGURE 2:**
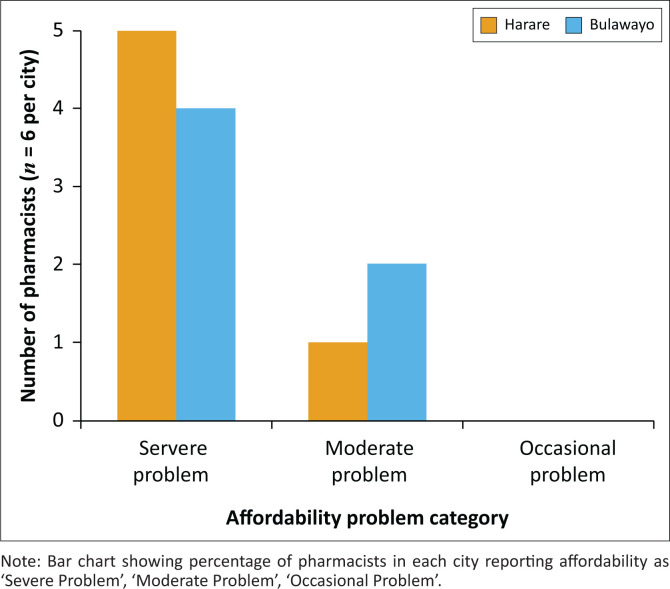
Comparative perceptions of affordability in Harare versus Bulawayo.

Taken together, these reflections illustrate that while Harare experiences are shaped more by market competition and rigid pricing systems, Bulawayo pharmacists navigate their dilemmas within more relational, community-embedded contexts, albeit with significant supply challenges. These contextual differences deepen understanding of how ethical pricing dilemmas manifest within Zimbabwe’s diverse urban settings. Figure 2 compares pharmacists’ perceptions of medicine affordability challenges between Harare and Bulawayo.

### Theme 4: Calls for systemic support

Across both cities, pharmacists highlighted the absence of formal guidance on ethical pricing and called for:

- National ethical pricing guidelines.
- Subsidy partnerships for chronic medicines.
- Professional peer forums to discuss dilemmas.

Pharmacists repeatedly emphasised the tension between business viability and patient welfare:

‘We need a framework that recognises our business realities but still ensures no one dies because they cannot afford medicines.’ (P12, Harare, 5 years)

Several participants expressed discomfort with the lack of national oversight:

‘Right now everyone prices the way they think is reasonable, but without guidelines we’re always unsure if we’re doing the right thing.’ (P04, Bulawayo, 9 years)

Others linked systemic support to improved consistency and fairness:

‘Patients compare prices across pharmacies and think we are cheating them, yet we don’t have a standard national reference point.’ (P14, Harare, 9 years)

A number of pharmacists stressed that subsidies for chronic conditions would protect vulnerable groups:

‘People with diabetes and hypertension suffer the most. If government could subsidise basics like insulin, it would change lives.’ (P06, Bulawayo, 4 years)

Peer support and platforms for collective reflection were also frequently mentioned:

‘We need regular forums where pharmacists can discuss difficult cases and agree on ethical boundaries, instead of working in isolation.’ (P15, Harare, 11 years)

Some also highlighted how systemic guidance could reduce moral distress:

‘Sometimes it feels like you’re choosing between helping a patient and keeping the pharmacy open. Clear guidelines would reduce that burden.’ (P06, Bulawayo, 4 years)

### Integrated insights with quantitative context

The ethical dilemmas described by pharmacists were reinforced by affordability data from a larger doctoral study conducted by the same author at the University of Zambia, Graduate School of Business.^12^ For instance, in Harare, median price ratios (MPRs) for commonly prescribed medicines were 6.4 compared to international reference prices, while in Bulawayo they averaged 5.8. Treatments for non-communicable diseases, such as insulin and antihypertensives, required between 3 days and 8 days’ wages of the lowest-paid worker, underscoring the moral weight of pharmacists’ decisions.

## Discussion

This study contributes to international qualitative work on pharmacy ethics by situating Zimbabwean pharmacists’ experiences within a broader discourse on moral dilemmas in professional practice. Previous studies in Europe, the Middle East, and Asia have documented that community pharmacists face significant ethical challenges when balancing business viability with patient care.^13,14,15^ Our findings resonate with this global evidence, while adding new insights from a fragile, hyperinflationary context where the absence of medicine price regulation amplifies these tensions.

From a health systems perspective, our findings mirror long-standing evidence that medicines in many LMIC private markets are frequently priced above international benchmarks, with affordability gaps greatest for chronic conditions. Standardised WHO/HAI surveys and pooled analyses have consistently shown high MPRs and heavy out-of-pocket burdens in settings where regulation is weak.^5,16^ In Zimbabwe, where over 70% of health expenditure is out-of-pocket, affordability constraints dominate patients’ access to medicines. Pharmacists reported recurrent scenarios of moral distress when patients could not afford prescribed therapies. Such experiences mirror international literature that links affordability barriers with compromised care and inequities. Local data reinforce this: the 2022 FinScope survey found that low-income households often forgo medicines entirely, while national expenditure reviews confirm the high financial burden of health care.^17,18^

A key contribution of this study is to foreground how pharmacists themselves respond within these constraints. Informal practices we observed, discounts, partial courses, and ad hoc credit, are ethically motivated but structurally fragile: they depend on individual discretion and business tolerance for loss, and they may unintentionally shift clinical risk to patients (e.g. sub-therapeutic courses). Similar tensions have been described in other settings where pharmacists’ professional values collide with reimbursement or pricing policies.

Our data also suggest city-level nuance. Harare pharmacists emphasised currency volatility and chain policies, whereas Bulawayo pharmacists emphasised community obligations and flexibility. This qualitative pattern aligns with Zimbabwe-focused pricing literature that reports high mark-ups, variability across outlets, and ongoing debate about regulation and transparency.^3,19,20,21^

The Zimbabwean experience also parallels broader sub-Saharan African patterns. A systematic review of pricing policies across the region concluded that weak regulatory frameworks perpetuate inequities and inefficiencies.^22^ In South Africa, the introduction of the Single Exit Price (SEP) policy substantially improved transparency and stabilised prices for both originator and generic medicines.^23,24^ While contexts differ, the South African example demonstrates how regulatory instruments can mitigate ethical dilemmas by reducing discretionary pricing power.

Beyond affordability, this study highlights the psychological burden on pharmacists who must reconcile their role as clinicians with commercial realities. The concept of ‘moral distress’ is well-established in health professions ethics, and recent work has begun to adapt and validate instruments for pharmacists.^14,25^ Our findings reinforce the need for such tools in Zimbabwe, not only to measure moral distress but also to inform targeted interventions, professional guidelines, and support systems.

Taken together, these findings underline that pharmacists are critical actors at the interface of commerce and care. Without systemic interventions such as fair pricing frameworks, professional support, and regulatory reform, the ethical burden will remain individualised, perpetuating both inequities in access and moral distress within the profession.

### Implications for policy and practice

Firstly, transparent pricing and consumer information can reduce search costs and opportunistic pricing. Zimbabwean work specifically argues for accessible price and availability information to support timely, affordable access.^21^

Secondly, mark-up control and reference pricing deserve serious consideration. Across sub-Saharan Africa, evidence syntheses describe mark-up regulation and international reference pricing as core tools to curb excess prices.^22^ South Africa’s SEP model is instructive: time-series analyses show immediate and sustained downward pressure on private-sector medicine prices after SEP introduction, with moderated annual increases thereafter.^23,24^ While Zimbabwe’s context differs, the directional evidence supports piloting regulated maximum mark-ups and/or ex-factory price controls for a priority basket (e.g. insulin, antihypertensives), alongside exemptions or risk-adjusted allowances to protect supply viability.

Thirdly, targeted financial protection (vouchers, means-tested subsidies, chronic disease schemes) is essential to mitigate the ethical bind at the counter. Broader fiscal analyses show that catastrophic health expenditure remains a risk for low-income households in Zimbabwe; any pricing reform should be paired with demand-side support.^17,18^

Fourthly, professional bodies can reduce moral distress by embedding ethics support (peer forums, case rounds) and practice guidance for pricing dilemmas (e.g. policies on partial dispensing, hardship discounts, and referral pathways). Instruments to measure moral distress among pharmacists have been developed and used to identify high-strain scenarios; these could be adapted locally to monitor well-being and evaluate interventions.^14,25^

### Strengths and limitations

Strengths include a focus on frontline ethical reasoning in two major urban markets and triangulation with sectoral evidence. Limitations include the qualitative sample size and urban scope (not generalisable to rural areas), potential social desirability bias, and the absence of direct price auditing within this article (although convergent evidence exists in the Zimbabwe literature).

### Future research

Priorities include mixed-methods evaluations of pricing reforms (e.g. capped mark-ups, reference pricing), pragmatic trials of price-transparency platforms, and moral-distress mitigation (ethics rounds, decision aids) in community pharmacy. Implementation research should track unintended effects (e.g. stock-outs or product withdrawal) observed in other regulated markets.

### Recommendations

- Develop National Ethical Pricing Guidelines:
  ■ The Medicines Control Authority of Zimbabwe (MCAZ), in collaboration with the Pharmaceutical Society of Zimbabwe, should formulate binding guidelines that define fair and ethical pricing practices. These should address mark-up caps, transparency requirements, and acceptable coping strategies when patients face financial barriers.
- Implement Transparent Mark-up Regulation:
  ■ Drawing from successful models such as South Africa’s SEP, Zimbabwe should consider piloting a regulated mark-up structure for essential medicines, starting with chronic disease treatments such as insulin and antihypertensives. Transparency in price components should be mandatory to improve accountability.
- Introduce Targeted Subsidy Mechanisms:
  ■ Public–private partnerships and donor collaborations should be leveraged to establish subsidy or voucher schemes for priority medicines. Such schemes would directly reduce out-of-pocket expenditure for low-income patients and alleviate pharmacists’ ethical burden at the counter.
- Strengthen Consumer Information Systems:
  ■ Affordable digital platforms should be established to provide real-time information on medicine prices and availability across pharmacies. Empowering patients with price transparency will reduce inequities and prevent exploitative practices.
- Embed Ethics Training and Support in Pharmacy Practice:
  ■ Pharmacy curricula and continuing professional development (CPD) programmes should include structured modules on pricing ethics, moral distress management, and patient-centred decision-making. Peer forums should be established where pharmacists can share dilemmas and solutions without fear of business consequences.
- Invest in Local Manufacturing and Supply Chain Resilience:
  ■ To reduce dependency on imports and foreign currency volatility, Zimbabwe should support local pharmaceutical manufacturing through tax incentives, preferential procurement, and streamlined regulatory processes. Strengthening local supply chains will stabilise pricing and availability.

### Implications for primary health care and family medicine

The findings of this study have direct implications for frontline PHC in Zimbabwe and comparable low-resource settings. Pharmacists are often the first and last point of contact for patients seeking medicines, particularly where public facilities face chronic stock-outs. Ethical dilemmas at the counter therefore translate into gaps in PHC delivery, undermining continuity of care for chronic diseases such as hypertension, diabetes, and HIV.

Strengthening medicine pricing regulation and supporting pharmacists with ethical guidance can improve equitable access to essential medicines, reduce catastrophic health expenditure, and enhance trust in community-based health services. For family medicine practitioners, improved collaboration with pharmacists is vital, as equitable access to prescribed treatments directly influences patient adherence, disease outcomes, and household well-being.

By addressing affordability through systemic reforms, including mark-up regulation, subsidy schemes, and enhanced information systems, Zimbabwe’s health system can better align with UHC goals and strengthen the foundations of PHC. Supporting pharmacists as integral PHC actors ensures that ethical tensions are resolved not at the individual level, but through a health system that prioritises patient welfare alongside sustainable pharmacy practice.

## Conclusion

Pharmacists in Zimbabwe’s private retail sector face profound ethical dilemmas in pricing life-saving medicines. The findings from Harare and Bulawayo highlight that moral distress is common, arising when patients cannot afford essential treatments. While pharmacists often adopt informal coping mechanisms such as silent discounts or partial dispensing, these practices are unsustainable and risk compromising patient care.

The study underscores the urgent need for systemic interventions rather than reliance on individual discretion. National ethical pricing guidelines, transparent mark-up regulation, and targeted subsidy mechanisms for chronic conditions are critical to safeguard patient access while maintaining pharmacy viability. Professional bodies should also provide structured forums and training to help pharmacists navigate ethical challenges without bearing the burden alone.

By giving voice to pharmacists, this research demonstrates that pricing decisions are not merely economic transactions but deeply moral encounters. Addressing medicine affordability in Zimbabwe requires both policy reform and professional support systems to ensure that no patient is denied treatment on the basis of cost.

## References

[1] World Health Organization, United Nations Children’s Fund (UNICEF). A vision for primary health care in the 21st century: Towards universal health coverage and the sustainable development goals [homepage on the Internet]. Geneva: WHO & UNICEF; 2018 [cited 2025 Aug 31]. Available from: https://iris.who.int/handle/10665/328065

[2] Cameron A, Roubos I, Ewen M, et al. Differences in the availability of medicines for chronic and acute conditions in the public and private sectors of developing countries. Bull World Health Organ. 2011;89(6):412–421. 10.2471/BLT.10.084327 21673857 PMC3099556

[3] Nakambale HN, Tambama P, Bangalee V. Regulation of mark-up on medicine prices in Zimbabwe: A pilot survey from 92 community pharmacies in the metropolitan area of Harare. Health Econ Rev. 2024;14(1):92. 10.1186/s13561-024-00574-8 39546124 PMC11566648

[4] Trap B, Hansen EH, Hogerzeil HV. Prescription habits of dispensing and non-dispensing doctors in Zimbabwe. Health Policy Plan. 2002;17(3):288–295. 10.1093/heapol/17.3.288 12135995

[5] Cameron A, Ewen M, Ross-Degnan D, Ball D, Laing R. Medicine prices, availability, and affordability in 36 developing and middle-income countries: A secondary analysis. Lancet. 2009;373(9659):240–249. 10.1016/S0140-6736(08)61762-6 19042012

[6] World Health Organization, Health Action International. Measuring medicine prices, availability, affordability and price components [homepage on the Internet]. 2nd ed. Geneva: WHO; 2008 [cited 2025 Aug 02]. Available from: https://www.who.int/publications/i/item/WHO-PSM-PAR-2008.3

[7] Masiye F, Kaonga O, Kirigia JM. Does user fee removal policy provide financial protection from catastrophic health care payments? Evidence from Zambia. PLoS One. 2016;11(1):e0146508. 10.1371/journal.pone.0146508 26795620 PMC4721670

[8] O’Donnell O, Van Doorslaer E, Rannan-Eliya RP, et al. Who pays for health care in Asia? J Health Econ. 2008;27(2):460–475. 10.1016/j.jhealeco.2007.08.005 18179832

[9] Dzinamarira T, Dzobo M, Chitungo I. COVID-19: A perspective on Africa’s capacity and response. J Med Virol. 2020;92(11):2465–2472. 10.1002/jmv.26159 32525568 PMC7300956

[10] Jamison DT, Summers LH, Alleyne G, et al. Global health 2035: A world converging within a generation. Lancet. 2013;382(9908):1898–1955. 10.1016/S0140-6736(13)62105-4 24309475

[11] Guest G, Namey E, Chen M. A simple method to assess and report thematic saturation in qualitative research. PLoS One. 2020;15(5):e0232076. 10.1371/journal.pone.0232076 32369511 PMC7200005

[12] Sibanda D. Analysis of factors determining the regulation of pricing of essential medicines in private retail pharmacies in Zimbabwe [doctoral thesis]. Lusaka: Graduate School of Business, University of Zambia; 2025.

[13] Kruijtbosch M, Göttgens-Jansen W, Floor-Schreudering A, Van Leeuwen E, Bouvy ML. Moral dilemmas of community pharmacists: A narrative study. Int J Clin Pharm. 2018;40(1):74–83. 10.1007/s11096-017-0561-0 29159520 PMC5840248

[14] Astbury JL, Gallagher CT. Development and validation of a questionnaire to measure moral distress in community pharmacists. Int J Clin Pharm. 2017;39(1):156–164. 10.1007/s11096-016-0413-3 28004238 PMC5306186

[15] Esmalipour R, Larijani B, Mehrdad N, Ebadi A, Salari P. The ethical challenges in pharmacy practice in community pharmacies: A qualitative study. Saudi Pharm J. 2021;29(12):1441–1448. 10.1016/j.jsps.2021.11.003 35002382 PMC8720823

[16] Wirtz VJ, Hogerzeil HV, Gray AL, et al. Essential medicines for universal health coverage. Lancet. 2017;389(10067):403–476. 10.1016/S0140-6736(16)31599-9 27832874 PMC7159295

[17] Reserve Bank of Zimbabwe, FinMark Trust, Zimbabwe National Statistics Agency (ZIMSTAT). FinScope Zimbabwe Consumer Survey 2022 [homepage on the Internet]. Harare: RBZ; 2022 [cited 2025 Aug 06]. Available from: https://www.rbz.co.zw/documents/BLSS/2022/Zimbabwe_FinScope_Consumer_2022_Survey_Report.pdf

[18] World Bank. Zimbabwe health sector public expenditure review [homepage on the Internet]. Washington, DC: World Bank; 2022 [cited 2025 Aug 14]. Available from: https://documents1.worldbank.org/curated/en/099730506292238494/pdf/P16556501fc556037089b80ca23e3977f6c.pdf

[19] Nakambale HN, Bangalee V. Global outsourcing and local tendering supply chain systems in the public healthcare sector: A cost comparison analysis, Namibia. Value Health Reg Issues. 2022;30:1–8. 10.1016/j.vhri.2021.09.008 34915421

[20] Lane J, Nakambale H, Kadakia A, et al. A systematic scoping review of medicine availability and affordability in Africa. BMC Health Serv Res. 2024;24(1):91. 10.1186/s12913-023-10494-8 38233851 PMC10792840

[21] Mureyi D, Gwatidzo SD, Matyanga CMJ. Consumers’ access to information about medicine prices and availability as an enabler of last mile medicine access: A scoping review. J Med Access. 2022;6:27550834221098598. 10.1177/27550834221098598 36204520 PMC9413504

[22] Koduah A, Gavor E, Nonvignon J, et al. Implementation of medicines pricing policies in sub-Saharan Africa: A systematic review. Syst Rev. 2022;11(1):216. 10.1186/s13643-022-02114-z 36457058 PMC9714131

[23] Moodley R, Suleman F. The impact of the single exit price policy on a basket of originator medicines in South Africa (1999–2014): Time-series analysis. BMC Health Serv Res. 2019;19(1):576. 10.1186/s12913-019-4419-2 31419977 PMC6697979

[24] Moodley R, Suleman F. The impact of South Africa’s single exit price on a basket of generic medicines: Time-series analysis. PLoS One. 2019;14(12):e0219690. 10.1371/journal.pone.0219690 31365534 PMC6668780

[25] Astbury JL, Gallagher CT. Moral distress among community pharmacists: Causes and achievable remedies. Res Social Adm Pharm. 2020;16(3):321–328. 10.1016/j.sapharm.2019.05.019 31171433

